# Late-Onset Angiotensin-Converting Enzyme Inhibitor-Induced Angioedema in a General Practitioner’s Practice: A Case Report

**DOI:** 10.3390/reports9020126

**Published:** 2026-04-20

**Authors:** Eva Jūlija Tirāne, Edgars Tirāns

**Affiliations:** 1 Faculty of Medicine, Rīga Stradiņš University, LV-1007 Rīga, Latvia; 2 Department of Family, Maternal and Child Health, University of Latvia, LV-1586 Rīga, Latvia; etirans@yahoo.com

**Keywords:** angiotensin-converting enzyme inhibitor, drug-induced angioedema, late-onset angiotensin-converting enzyme inhibitor-induced angioedema, perindopril

## Abstract

**Background and Clinical Significance:** Angiotensin-converting enzyme inhibitors (ACE-Is) are commonly used for treatment of hypertension and are well known among primary care specialists. ACE-I-induced angioedema is a rare, yet possible side effect. It should not be taken lightly, as it can be life-threatening. It is characterized by erythematous or skin-coloured, self-limiting, localized, non-pitting swelling of the submucosal and/or subcutaneous layers of tissue. Usually, it develops in the first year of using the medication, although it can also start several years after using it. Herein, we describe a late-onset ACE-I-induced angioedema, which developed 7 years after using the ACE-I. This case report depicts the challenges of diagnosing ACE-I-induced angioedema, especially if it is late-onset. It highlights the importance of actively asking patients questions about possible side effects of medication even several years after using it and the patients themselves not having any complaints. **Case Presentation:** We present a 61-year-old Caucasian male with recurring swelling of the lips, tongue and an uncomfortable feeling in the throat, which started 7 years after using an ACE-I: perindopril. There was no airway obstruction or urticaria in any of the episodes. Hereditary angioedema was ruled out by blood analysis. Based on the clinical presentation, images and blood analysis, it was diagnosed as late-onset ACE-I-induced angioedema. After discontinuing the ACE-I, there were two more episodes of angioedema reported, which were a lot milder in symptoms and lasted a shorter time period. Since then, there have been no other episodes of angioedema. **Conclusions:** It is important to keep in mind angioedema as a possible side effect for patients on ACE-Is. Patients should be regularly and actively questioned about side effects, even if the medication has been started several years ago and no complaints are brought up by the patient.

## 1. Introduction and Clinical Significance

Angiotensin-converting enzyme inhibitors (ACE-Is) are commonly prescribed medications. They are frequently used for many diseases, for example, hypertension and chronic heart failure. ACE-Is can also be used in combination with other drugs, such as diuretics and beta-blockers [1].

One of the most common side effects of ACE-Is includes a dry, nonproductive, and irritating cough, which can be easily spotted by patients and doctors alike. Another possible side effect is angioedema. It is also known as angioneurotic edema and is a potentially life-threatening complication [2]. Angioedema is a localized, self-limiting, non-pitting swelling of the submucosal and/or subcutaneous layers of tissue, which can be erythematous or skin-coloured [2,3]. The overall incidence of angiotensin-converting enzyme inhibitor (ACE-I)-induced angioedema is 0.49% [4]. At a higher risk are women, patients on non-steroidal anti-inflammatory drugs, and African Americans [4]. Mostly angioedema develops within the first year of starting an ACE-I, although it can also occur several years after starting the medication [4,5].

Angioedema is characterized by a rapidly increasing vascular permeability and following edema [6]. It can last from a few hours up to several days [2,7]. Angioedema can have various localisations. Commonly affected areas are lips, mouth, tongue, neck, larynx, extremities, gut, and genitalia [3,7].

The most crucial step of treatment in case of ACE-I associated angioedema is discontinuation of the medication. Another is ensuring an adequate airway. In severe cases, patient management in the intensive care unit with tracheostomy or endotracheal intubation may be necessary [8,9]. Antihistamines, corticosteroids, and epinephrine are mostly ineffective. Bradykinin receptor antagonists, such as icatibant, and kallikrein inhibitors, such as ecallantide, are not supported in randomized trials. There have been reports of reduced symptom duration in several case studies using fresh frozen plasma and C1-inhibitor (C1-INH) concentrate; however, this has not been tested in trials [10].

We present a case of a 61-year-old Caucasian male with late-onset ACE-I-induced angioedema in a general practitioner’s practice. Hypertension can be treated by a general practitioner (GP), and ACE-Is are a commonly used treatment option. Thus, general practitioners should consider the rare yet possible side effect of this group of medications—angioedema. The diagnostic process can be relatively difficult due to the clinical presentation. In this case, the patient thought the symptoms to be linked with oral herpes infection or an allergic reaction, and thus did not go to the GP with complaints earlier. It should be a routinely asked question if the patient has experienced swelling of the submucosal and/or subcutaneous tissue while taking an ACE-I, even if they have not expressed any complaints and the medication had been used for several years before.

## 2. Case Presentation

A 61-year-old Caucasian male presented to the GP with complaints of recurring swelling of the lips and tongue. The episodes had started 6 years ago, with each episode lasting from 24 to 48 h. The last episode that caused the patient to see the GP lasted two days and included swelling of the lips, tongue, and one-sided swelling of the genitalia. The patient had a medical history of arterial hypertension (diagnosed and treatment started in 2012), chronic hepatitis C (diagnosed in 2003, successfully treated in 2015 with complete elimination of virus, PCR (RNA)-negative), lower extremity phlebitis and thrombophlebitis (2018), and oral herpes infection (onset in 1979). There was no family history of angioedema, swelling, or drug allergies. The patient had no history of drug reactions, food allergies, dyspnea, or severe abdominal pain. The patient had started antihypertensive treatment in 2012, using a combination of perindopril 5 mg and indapamide 1.25 mg daily. In 2014, the dose was increased to 8 mg of perindopril and 2.5 mg of indapamide daily. In 2021, due to ineffectiveness, the medication was changed to 8 mg of perindopril and 5 mg of amlodipine daily. In 2024, the dose was increased to 8 mg of perindopril and 10 mg of amlodipine daily. There were no other medications that the patient took regularly. The patient had previously expressed no side effects of the treatment.

On presentation at the general practitioner’s office, the patient’s blood pressure was 125/80 mmHg, heart rate was 60 bpm, temperature was 36.6 °C, oxygen saturation 98%, and respiratory rate was 16 breaths/minute. He had no signs of swelling of the submucosal or subcutaneous tissue anywhere in the body. The patient came into the GP’s office with complaints of recurring swelling of the lips, tongue, and an uncomfortable feeling in the throat. The first probable episode of it was six years ago in 2019, when, after eating a steak, the patient experienced swelling of the tongue. It was mild and lasted 8 h, not causing too much of a discomfort. There was no involvement of any other tissue localisation, no urticaria, nor airway obstruction. He thought it was the result of biting himself on the tongue while eating, therefore no medical attention was sought. After this, the episodes of angioedema recurred.

**Figure 1:**
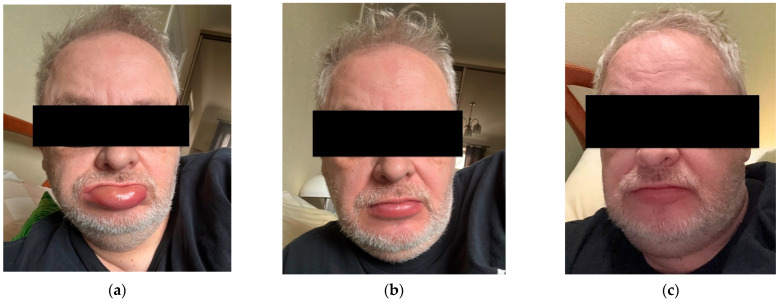
Photographs showing lip swelling throughout an episode within a day: (**a**) swelling of the lower lip at 11:30; (**b**) swelling of the lower lip at 14:00, same day; (**c**) swelling of the lower lip at 17:20, same day.

In the first few episodes, the swelling affected only the tongue, but afterwards it started to involve the lips and larynx. The episodes usually lasted from 24 to 48 h, with visible changes in the localisation of the swelling of the lips. There was no pattern to when the incidents would occur. The patient admitted that in the last two years, the episodes were more frequent, occurring four to six times per year. The initial symptoms were an uncomfortable feeling in the throat, probably caused by the swelling of the larynx, gradually causing mild dysphagia during some episodes. After that came a tingling sensation and asymmetric swelling of the lips, initially affecting one lip and, after a few hours, affecting the other. In any of the episodes there was no urticaria or any type of skin involvement, nor airway obstruction. The last episode involved one-sided swelling of the genitalia, which was not present in any of the previous events. As a result of that, he made the decision to go to the GP’s office, despite the fact he did not bring these episodes up in any of his other previous doctor’s visits. Prior to it the patient thought that the swelling was an allergy, thus using antihistamines, although there was minimal to no result. The patient did not observe any link between a food item or allergen and symptoms of angioedema. Due to the tingling sensation of the lips, the patient thought the symptoms to be linked to the prodromal phase of oral herpes infection, therefore acyclovir was used; however, it was ineffective in reducing the symptoms. During the consultation with the GP, it was revealed that there were no other symptoms of oral herpes infection, for example, no blisters around, on, or inside the mouth, while there were signs of angioedema. In other situations, where the patient had an episode of oral herpes infection, the patient reported blisters around the mouth, which were not present in any episodes of the angioedema. The patient showed the general practitioner photographs that he had taken of the swelling of the lips throughout an episode (Figure 1).

After the consultation at the GP’s office, ACE-I-induced angioedema was suspected on clinical suspicion. Perindopril was discontinued. The patient was initiated on hydrochlorothiazide 25 mg and amlodipine 10 mg. Blood analysis was taken to rule out hereditary angioedema and acquired angioedema secondary to antibodies against C1-inhibitor (C1-INH). The results showed C4, C1-INH activity, C1-INH concentration, and C1q antibodies within the normal range (Table 1).

Based on the symptoms and blood analysis, this was diagnosed as late-onset ACE-I-induced angioedema. A few days after changing the antihypertensive treatment, there were two episodes of mild one-sided swelling of the tongue. These episodes lasted a few hours each, subsiding spontaneously, and did not cause any discomfort to the patient. Since then, there have been no other episodes. The patient kept using the changed treatment.

## 3. Discussion

This case report describes a late-onset angiotensin-converting enzyme inhibitor-induced angioedema in a general practitioner’s practice. While quite uncommon, ACE-Is can cause angioedema, as shown in the images taken by the patient. As in this case, the angioedema started several years after initially starting the medication and it was more difficult to diagnose. The diagnostic process was also made more complicated due to the fact that the patient never went to the GP with any complaints of swelling in any localization, by the reason of suspicions of a different etiology.

Angioedema is defined as an erythematous or skin-coloured swelling that is self-limiting, localized, and non-pitting, affecting the submucosal and/or subcutaneous layers of tissue [2,3]. In a case–control study from 2008 to 2018, it was estimated that the incidence of both early and late-onset angioedema in patients using an ACE-I is 0.49%, with the median age being 66 years [4]. Notably, the patient in this case report had no risk factors of ACE-I-induced angioedema, like nonsteroidal anti-inflammatory drug use [4]. In the study it was found that most of the patients (34%) developed angioedema within the first year of using an ACE-I, while only 11% developed angioedema within the first month of starting the medication [4]. It is important to note that, although less common, the first episode of angioedema can take place several years after starting the medication, as it was seen in this case [5].

There are many different types of angioedema. According to the DANCE classification (definition, acronyms, nomenclature, and classification of angioedema) angioedema can be divided into five types: mast-cell mediated angioedema, bradykinin-mediated angioedema, angioedema due to vascular endothelium dysfunction, drug-induced angioedema, and angioedema due to unknown cause [11]. Each of the aforementioned categories has a different pathophysiologic mechanism. In the case of mast-cell mediated angioedema, the underlying mechanism is mast cell degranulation. Angioedema that is bradykinin-mediated can have many causes: acquired C1-INH deficiency, hereditary C1-INH deficiency, and kallikrein-kinin system pathway mutations. If there is impaired function of the intrinsic endothelium, it is classified as angioedema due to vascular endothelium dysfunction. In the case of drug-induced angioedema, there are many suspected mechanisms, all involving drug adverse reactions, including the side effects of ACE-Is, as seen in this case report. If the etiology is not known, it is classified as angioedema due to unknown causes [11].

It was essential to rule out a possible diagnosis of hereditary angioedema. Hereditary angioedema is a rare genetic disorder that is caused by a dysfunction or deficiency of the C1-INH, although in some cases the functional and quantitative levels of C1-INH are normal [12]. Patients affected by hereditary angioedema typically present with symptoms in childhood or young adulthood, usually aggravating around puberty [13]. The episodes of angioedema generally take place earlier in the case of the hereditary form of angioedema than in the acquired [12]. As it is an autosomal-dominant disorder, children of a parent with hereditary angioedema have a 50% chance of inheriting the condition. In childhood, episodes are milder and intermittent, mostly causing pain in the abdomen; however, later in life, symptoms usually worsen [14]. Hereditary angioedema typically presents with noticeable swelling of the face, oropharyngeal structures, and extremities, albeit it can affect other localisations [12]. Even though the episodes mostly have a spontaneous onset, in some cases, there are triggers, for example, mental stress, surgical procedures, physical stress, or infections. Typically, episodes last 3–5 days before resolving spontaneously [15]. There are three main types of hereditary angioedema. Type I presents with C1-INH deficiency, and it is the most common form of hereditary angioedema. Type II is characterized by a dysfunctional C1-INH. The last type is hereditary angioedema with normal C1-INH, formerly known as type III, which is the least common form [11,14]. Laboratory testing is used to exclude or confirm the diagnosis of hereditary angioedema. Type I is diagnosed when the C1-INH function and concentration are both below the normal range. Type II is diagnosed when the C1-INH function level is below the normal range, although the concentration is normal or high. Low C4 level supports a diagnosis of hereditary angioedema [16]. If the patient has acquired angioedema in the result of acquired C1-INH deficiency (due to antibodies generated against C1-INH), the C4 is low, C1-INH levels are normal or low, C1-INH function is low, and C1q levels are low as well [15].

Angioedema due to unknown causes, i.e., idiopathic angioedema, is another subtype of angioedema. The main characteristic of this type of angioedema is that the etiology and mechanism is not known [11]. It can be difficult to differentiate from ACE-I-induced angioedema. One of the aspects that distinguish the two of them is time period. ACE-I-induced angioedema should improve after stopping the medication, with some episodes continuing for up to six months [17]. There can be an overlap, where the diagnosis is not clear, so it is important to observe if the patient has episodes of angieodema that continue after the aforementioned time period. Some patients that have received ACE-I or angiotensin II receptor blocker therapy and experienced episodes of angioedema can have recurrent episodes of angioedema even after stopping the use of medication for more than half a year, proving the initial diagnosis wrong [18]. In a study between 2007 and 2018, it was found that only 59% of the patients were correctly diagnosed with ACE-I or angiotensin II receptor blocker-induced angioedema, while 41% of them had a different cause of angioedema [18]. This stresses the importance of observing the patient carefully, as the episodes of angioedema should eventually cease after stopping the medication, which has been linked to the cause of symptoms. Idiopathic angioedema is suspected when there is no identifiable cause of it, while the use of ACE-Is, without any other etiology of the symptoms, suggests ACE-I-induced angioedema [18]. Idiopathic angioedema usually presents without urticaria [19]. Patients can be referred to as having idiopathic angioedema, while simultaneously continuing investigations to find out the underlying cause. Idiopathic angioedema can be histaminergic or non histaminergic. In most cases it seems to be responsive to antihistamines, also differentiating it from ACE-I-induced angioedema [19].

The main target of ACE-Is is the renin–angiotensin–aldosterone system (RAAS). It decreases angiotensin II synthesis and causes a decrease in the secretion of aldosterone and decreased activity of vasopressin, leading to cardiovascular and antihypertensive effects. The kallikrein-kinin system is also a part of the mechanism, being the antagonist of RAAS. Kallikrein acts as a protease, converting high-molecular-weight kininogens into kinins, mainly bradykinin. Bradykinin acts as a vasodilator by binding to bradykinin receptor B_2_, mediating the release of prostacyclin, substance P, nitric oxide, and endothelium-derived hyperpolarizing factor [5]. These substances increase vascular permeability, resulting in the characteristic symptoms of angioedema-localized inflammation and edema. In a normal setting, angiotensin-converting enzyme would degrade bradykinin. ACE-I usage results in a decreased metabolism of bradykinin, which can lead to the characteristics of angioedema. It is important to note that not every patient on ACE-I develops angioedema, indicating that other factors could be involved. For example, an association between the BDKRB2 variant has been linked to an increased risk of angioedema in patients receiving ACE-I treatment. Other risk factors include a history of seasonal allergy, smoking, use of immunosuppressive medication, previous drug-induced rash, and ethnicity [5]. In this case report, the patient had none of the aforementioned risk factors.

In the early stages, angioedema can be easily missed due to nonspecific symptoms affecting the lips, tongue, and mouth, which can suggest that the cause is food or other medication [2]. The areas affected can also be the neck, extremities, larynx, gastrointestinal tract, and genitalia [3,7]. Symptoms of ACE-I-induced angioedema usually last 48–72 h [6]. If the larynx is involved, it can be life-threatening, while gut involvement can cause severe pain, mimicking acute abdominal conditions [7].

Finding a correct diagnosis in the case of angioedema is not easy. Initially, the main diagnostic tool is clinical diagnosis, as in many cases, patients with angioedema need immediate intervention and laboratory methods are time-consuming [20]. Laboratory tests should be taken to confirm the diagnosis. In case of drug-induced angioedema, C4, C1-INH concentration, C1-INH function and anti-C1q antibodies are all within the normal range, as also seen in this case report [17,20].

The most important treatment of ACE-I-induced angioedema is discontinuation of the drug responsible for the adverse reaction [20]. After stopping the medication, the episodes of angioedema typically end; however, they can continue to occur up to 6 months after cessation of the ACE-I [17]. In this case report, the ACE-I was immediately discontinued after suspecting ACE-I-induced angioedema, and antihypertensive therapy was changed to hydrochlorothiazide and amlodipine, and only a few much milder episodes were observed by the patient afterwards. There is no specific therapy for ACE-I-induced angioedema. The standard treatment consists of glucocorticoids, epinephrine, and H1 antihistamines, although they may be ineffective. Since this type of angioedema is not histamine-mediated, antihistamines are mainly ineffective [20]. Due to the possibility of laryngeal angioedema, breathing and airway patency should be monitored closely [20]. Endotracheal intubation or surgical airway may be necessary [8]. Several drugs for hereditary angioedema have also been tested for ACE-I-induced angioedema. One of them is icatibant, which prevents the binding of bradykinin to its receptor. Early reports of it showed promising results of resolution or time to relief; however, this was not supported in larger randomized-controlled trials. Another drug used in treating hereditary angioedema is a kallikrein inhibitor called ecallantide. In general, it has not been proven to be effective in ACE-I-induced angioedema, although its efficacy in severe attacks is still under discussion [21]. C1 inhibitor may also be used in the treatment of ACE-I-induced angioedema. There have been several case reports confirming successful treatment of ACE-I-induced angioedema, although there are no prospective trials yet [22]. Fresh frozen plasma has also been used to treat patients with ACE-I-induced angioedema. In many cases, the treatment has been effective, resulting in symptom improvement or the stopping of progression. Nonetheless, no double-blind placebo-controlled trials have been yet performed to confirm its effects relative to placebo [21].

## 4. Study Limitations

This case report comes with some limitations. Although the episodes ceased, two more mild ones occurred shortly after stopping the medication. During the last month and a half there have been no episodes, which supports the diagnosis of it being ACE-I-induced angioedema. Nonetheless, it is not possible to fully rule out the origin of angioedema being some other factor. Although oral herpes infection can be the cause of angioedema, it is not likely in this case, as the patient reported no other characteristic symptoms of the infection at the time of the episodes and treatment for it was not effective in alleviating the angioedema. An allergy may be another possible etiology for this patient’s angioedema, although he did not find any specific food item, dye or additive to be linked to the symptoms. For example, the first probable episode of angioedema was after eating steak; nonetheless, the patient has had steak on other occasions without it causing any of the symptoms or discomfort. Furthermore, antihistamines were not effective. Due to ACE-I-induced angioedema being a diagnosis of exclusion, the patient’s symptoms may also be classified as angioedema due to unknown causes (idiopathic angioedema), although the usage of an ACE-I suggests otherwise. Furthermore, after ceasing the medication, the episodes of angioedema stopped.

## 5. Conclusions

This case highlights the rare, yet possible side effect of ACE-Is: angioedema. It demonstrates the difficult diagnostic process and the importance of ruling out hereditary angioedema. The most important step in case of ACE-I-induced angioedema is discontinuing the medication. This case report shines light on the importance of regularly and actively asking patients about the side effects of their medication, even if it has been used for several years with no complaints.

## Figures and Tables

**Table 1:** Blood analysis results.

Laboratory Tests	Patient Value	Reference Range
C4, g/L	0.31	0.12–0.36
C1 inhibitor concentration, g/L	0.35	0.21–0.38
Anti-C1q Antibodies	1.1	0–9.9
C1 inhibitor Activity, %	112	70–130

## Data Availability

The original contributions presented in the study are included in the article; further inquiries can be directed to the corresponding author.

## Abbreviations

The following abbreviations are used in this manuscript:

ACE-I	Angiotensin-converting enzyme inhibitor
ACE-Is	Angiotensin-converting enzyme inhibitors
C1-INH	C1 inhibitor
GP	General practitioner
RAAS	Renin–angiotensin–aldosterone system
